# Visual acuity changes in idebenone treatment‑naïve patients with Leber hereditary optic neuropathy: insights from the CRS-2 study

**DOI:** 10.1007/s00415-026-14181-6

**Published:** 2026-10-28

**Authors:** Maciej R. Krawczynski, Judith van Everdingen, Bart P. Leroy, Christophe Verny, Nöel J. C. Bauer, Cécile Andris, Birgit Lorenz, Marcela Votruba, Claudia Priglinger, Neringa Jurkute, Xavier Llòria, Magda Silva, Valerio Carelli, Patrick Yu-Wai-Man, Thomas Klopstock

**Affiliations:** 1 Department of Medical Genetics, Poznan University of Medical Sciences, 60-806 Poznan, Poland; 2 GENESIS - Centers for Medical Genetics, 60-529 Poznan, Poland; 3 Department of Neuro-Ophthalmology, The Rotterdam Eye Hospital (OZR), 3011 BH Rotterdam, The Netherlands; 4 The Rotterdam Ophthalmologic Institute (ROI), 3011 BH Rotterdam, The Netherlands; 5 Department of Ophthalmology & Center for Medical Genetics, Ghent University Hospital, Ghent, Belgium; 6 Department of Head & Skin, Ghent University, Ghent, Belgium; 7 Centre de Référence des Maladies Neurogénétiques, Département de Neurologie, CHU d’Angers, Angers, France; 8 MitoLab Team, UMR CNRS 6015 - INSERM U1083, Institut MitoVasc, SFR ICAT, Université Angers, Angers, France; 9 University Eye Clinic Maastricht, Maastricht University Medical Center, Maastricht, Netherlands; 10 CHU Sart Tilman, Liege, Belgium; 11 Department of Ophthalmology, Justus-Liebig-University and Universitätsklinikum Giessen and Marburg GmbH, Giessen Campus, Giessen, Germany; 12 Eye Department, Cardiff & Vale University Health Board, University Hospital of Wales, Cardiff, UK; 13 School of Vision Science, Cardiff University, Cardiff, UK; 14 Department of Ophthalmology, University Hospital, LMU Munich, Munich, Germany; 15 Moorfields Eye Hospital NHS Foundation Trust, London, UK; 16 National Hospital of Neurology and Neurosurgery, University College London Hospitals, London, UK; 17 Institute of Ophthalmology, University College London, London, UK; 18 Chiesi Farmaceutici SpA, Parma, Emilia-Romagna Italy; 19 Magda Joana Silva Lda, Porto, Portugal; 20 IRCCS Istituto di Scienze Neurologiche di Bologna, Programma di Neurogenetica, Bologna, Italy; 21 Department of Biomedical and Neuromotor Sciences, University of Bologna, Bologna, Italy; 22 Moorfields Eye Hospital NHS Foundation Trust, London, UK; 23 John van Geest Centre for Brain Repair and MRC Mitochondrial Biology Unit, Department of Clinical Neurosciences, University of Cambridge, Cambridge, UK; 24 Cambridge Eye Unit, Addenbrooke’s Hospital, Cambridge University Hospitals, Cambridge, UK; 25 Institute of Ophthalmology, University College London, London, UK; 26 Department of Neurology, Friedrich-Baur-Institute, LMU University Hospital, LMU Medizin, Ludwig-Maximilians-Universität (LMU) München, 80336 Munich, Germany; 27 German Center for Neurodegenerative Diseases (DZNE), 81377 Munich, Germany

**Keywords:** Leber hereditary optic neuropathy, Natural history, Mitochondrial disease, Optic neuropathy, Disease progression

## Abstract

**Objective:**

Case Record Survey (CRS)-2 was an international, multicenter, retrospective study designed to characterize the natural history of vision loss and spontaneous recovery in patients with Leber hereditary optic neuropathy (LHON).

**Methods:**

Case records of idebenone treatment-naïve patients aged ≥12 years, with mitochondrial DNA (mtDNA) variants m.3460G>A, m.11778G>A or m.14484 T>C, onset of symptoms (OoS) after 1999, and ≥2 visual acuity (VA) assessments within 5 years of OoS were analyzed. Disease phases were subacute/dynamic (≤1 year) or chronic (>1 year from OoS). Separate analyses were performed for eyes with VA assessments within 12 or 24±3 months from baseline (first VA assessment after OoS in each eye).

Outcomes included: spontaneous clinically relevant recovery (sCRR; improvement from off-chart to ≤1.6 logMAR, or ≥0.2 logMAR if on-chart), sCR stabilization (sCRS; maintenance of VA <1.0 logMAR), sCR benefit (sCRB; eyes with a sCRR and/or sCRS), and sCR worsening (sCRW; worsening to off-chart, or loss of ≥0.2 logMAR if on-chart). The primary endpoint was the proportion of subacute/dynamic eyes 12 months from baseline with sCRB.

**Results:**

Of 402 eligible eyes, 385 were subacute/dynamic at baseline, with 96 included in the 12-month analysis. Most patients were male (79.9%), with the m.11778G>A variant (71.7%). The primary endpoint was observed in 20.8% (n=20/96) of eyes. Spontaneous visual outcomes varied by disease phase, mtDNA variant, sex, and age at OoS.

**Conclusions:**

CRS-2 provides insights into LHON natural history and suggests the disease course may be more dynamic than previously reported, with spontaneous VA changes occurring well into the chronic phase.

**Trial registration:**

ClinicalTrials.gov NCT02796274, May 2016.

**Supplementary Information:**

The online version contains supplementary material available at https://doi.org/10.1007/s00415-026-14181-6.

## Introduction

Leber hereditary optic neuropathy (LHON) is an inherited mitochondrial disorder that leads to subacute, bilateral, painless vision loss [1, 2]. LHON prevalence ranges from approximately 1 in 31,000–68,000 in estimates from Northern England [3], Finland [4], The Netherlands [5], Denmark [6], Australia [7], and Japan [8]. Patients with LHON typically present with subacute central vision loss in one eye, with bilateral eye involvement within a matter of weeks or months [2, 9]. Symptom onset typically occurs between 15 and 35 years of age, with a male predominance of 80–90% [10], although a recent study reported that this ratio is closer to 1:1 at ages associated with lower estrogen levels (<5 and >45 years of age in female patients) as estrogen may act as a protective factor [11, 12]. Current understanding of the disease course describes most LHON patients experiencing severe decline in their visual acuity (VA) immediately after symptom onset, reaching a nadir at around 6 months [1]. After approximately a year, patients enter a chronic phase where vision loss is usually severe and permanent [1].

Over 90% of LHON cases are caused by one of three variants in the mitochondrial genome (m.3460G>A in *MT-ND1*, m.11778G>A in *MT-ND4*, m.14484T>C in *MT-ND6*) [13]. These variants affect subunits of complex I in the mitochondrial respiratory chain [14] and result in an energy production deficit and reactive oxygen species (ROS) accumulation [15, 16]. The resulting deficiency in energy impairs axonal transport, and the excess ROS may lead to retinal ganglion cell death [17–20].

Recent years have seen the therapeutic management of LHON evolve. Idebenone (Raxone^®,^ Chiesi Farmaceutici S.p.A., Italy), an orally administered structural derivative of ubiquinone, was approved in the European Union (EU) in 2015 [1] followed by several non-EU countries around the world for the treatment of LHON and remains the only available therapy for these patients. Although the pivotal, placebo-controlled, randomized, RHODOS study did not meet its primary endpoint, secondary endpoints demonstrated stabilized and improved VA in LHON patients treated within 5 years of symptom onset [21], with later studies confirming the benefit of long-term treatment [22–24]. Gene therapy is currently being studied as another potential approach to LHON management. Intravitreal injection of a recombinant adeno-associated viral vector (rAAV2) encoding human wild-type *MT-ND4* in patients with the m.11778G>A variant has shown promising results [25–27]. Unexpectedly, single unilateral injection of rAAV2 led to bilateral VA improvements, posing a challenge in assessing the therapeutic effect of the treatment, suggesting the need for further studies [25, 26].

Efficacy studies for LHON therapies are complicated by both the common challenge of obtaining control data in a rare disease, and the occurrence of spontaneous visual recovery [28]. Although VA loss in LHON often progresses to severe, permanent levels, some patients experience stabilization or spontaneous partial improvement in their vision [1]. The causative genetic variant is thought to be the most significant prognostic factor, followed by age at symptom onset [1, 29].

Previous natural history analyses have been limited by their small sample sizes, with variations in reported rates of spontaneous recovery, largely arising from inconsistent definitions of VA recovery or improvement, as well as differences in assessment timelines [3, 28, 30, 31]. Inclusion of patients who received unspecified treatment was also noted as a possibility in some of these analyses, therefore confounding the true rates of spontaneous recovery [28].

The case record survey (CRS)-2 study was designed to collect up-to-date retrospective patient data. Along with the earlier CRS-1 study, a separate case record survey to CRS-2 [24, 32, 33], it served as a repository of data on idebenone treatment-naïve eyes for the natural history control group that was matched and compared with treated eyes in the LEROS study [24]. CRS-2 also sought to clarify the natural history of vision loss and spontaneous recovery in patients with genetically confirmed LHON.

## Methods

### Study design

The CRS-2 study (NCT02796274) was an international, multicenter, retrospective, observational medical record survey conducted at 20 sites across seven countries (Belgium, France, Germany, Italy, the Netherlands, Poland and the United Kingdom) in Europe. Case records from 1999–2018 were reviewed, and data collection occurred from May 2016–March 2018. Data collected included demographics, relevant medical and LHON-specific history where available, all VA results, method or eye chart used (e.g., Early Treatment Diabetic Retinopathy Study [ETDRS], Snellen, and off-chart VA notations [ability to count fingers, see hand motion, have light perception and no light perception]) for VA at each assessment, as well as any idebenone use. The study was conducted according to the Guidelines for Good Pharmacoepidemiology Practices issued by the International Society for Pharmacoepidemiology. The independent ethics committees for each participating site that reviewed and approved the study protocol are provided in Table S1. A signed data release agreement form was obtained from all patients prior to data collection.

### Study population

CRS-2 inclusion criteria for patients were those aged ≥12 years at the time of data collection from case records, who had one of the three most common mitochondrial DNA (mtDNA) pathogenic variants (m.3460G>A, m.11778G>A, m.14484T>C), experienced symptom onset after 1999 with the month of onset known for both eyes, and had at least two VA assessments with defined dates available within 5 years of onset and prior to any idebenone use. Patients who participated in an interventional clinical trial after symptom onset, or who had any other cause of visual impairment or any active ocular disorder during the observation period were excluded. Case records from all patients fulfilling the above criteria were collected from each participating site. Patients from CRS-1 (NCT01892943) were included if they fulfilled the above criteria and the VA assessment after the last assessment reported in CRS-1 could be obtained.

### Definitions

Dates of symptom onset were recorded by the Investigator, with no formal criteria applied. For patient-level analyses, baseline was the first VA assessment after first symptom onset. For eye-level analyses, baseline was the first VA assessment after symptom development in that eye. Disease phase was defined by time since symptom onset at baseline; subacute/dynamic (≤1 year) and chronic (>1 year). VA blindness categories for eyes were as follows: non-legally blind: logMAR <1.0; legally blind: 1.0≤ logMAR ≤1.68; off-chart: logMAR >1.68. Sequential onset was defined as a difference in the number of months since onset of symptoms in the first versus the second eye; simultaneous onset was defined as no difference in the number of months between onset in the first and the second eye.

Four VA responses were used to describe the spontaneous (i.e., without idebenone treatment) achievement of outcomes for individual eyes from baseline to the specified timepoint, in line with outcome definitions used in the LEROS study [24]. Spontaneous clinically relevant recovery (sCRR) was defined as an improvement from baseline from either an off-chart VA to reading at least 5 letters on-chart (≤1.6 logMAR), or an improvement of at least 10 additional letters (−0.2 logMAR) for those already on-chart. Spontaneous clinically relevant stabilization (sCRS) was defined as maintenance of a baseline VA <1.0 logMAR. Spontaneous clinically relevant benefit (sCRB) was a composite measure that included eyes with a sCRR, sCRS, or both; if an eye had both, it was counted once for sCRB. Spontaneous clinically relevant worsening (sCRW) was defined as a worsening from baseline VA on-chart to off-chart or a loss of at least 10 letters (+0.2 logMAR) on-chart. For sCRS and sCRW, only those eyes eligible at baseline for these definitions were used to calculate the proportion, e.g., for sCRS, only those that were <1.0 logMAR at baseline were assessed.

### Outcomes

The primary endpoint of CRS-2 was designed to provide a comparator group of idebenone treatment-naïve patients to those treated with idebenone in the LEROS study and was defined as the proportion of subacute/dynamic eyes with sCRB at the 12-month assessment. See Supplemental Appendix I for a full description of secondary endpoints. Additional post hoc endpoints included changes in VA and changes in blindness categories.

### Statistical analyses and imputations

No formal sample size calculation was defined, based on the retrospective data collection study design. To standardize VA data, any VA values not originally in logMAR were converted using standard methods. Any off-chart logMAR value >1.68 (including VA assessments based on “counting fingers”, “hand motion”, “light perception”, “no light perception”) was assigned a value of 1.8 logMAR in order to standardize VA data from different physicians and to remain consistent with the LEROS study [24].

To remain consistent with the design of the LEROS study, analyses were performed on a per-eye basis because, according to previous reports, the majority of LHON patients do not develop symptoms in both eyes simultaneously [34], and not every visit had a VA assessment recorded in both eyes of the patient [24]. To facilitate subgroup comparisons of VA outcomes, eyes were identified with similar follow-up durations and those with VA assessments around 12 or 24 months after their baseline visit were selected. Specifically, eyes with assessments 12±3 months post-baseline were grouped for the 12-month analysis, and those with assessments 24±3 months post-baseline were grouped for the 24-month analysis.

Descriptive analyses were performed for eyes with similar follow-up durations, i.e., those with a 12- or 24-month assessment. Subgroup analyses were performed for LHON mtDNA variant, sex, and age at symptom onset. Age categories were selected based on the current understanding of LHON, reflecting early onset (<15 years), the typical age of onset (15–35 years), later than typical onset (>35 years), and ages at which estrogen levels are generally lower in females (<15 years and >50 years). Linear regressions on change in VA from baseline to the 12- or 24-month assessment were performed. SAS software (version 9.4; SAS Institute, Cary, NC, USA) was used for statistical analysis.

## Results

### Study population and baseline characteristics

In total, 219 patients were identified, 10 of whom had also participated in CRS-1. Two-thirds (67%) of visits occurred in or after 2010, the year of completion of the RHODOS study, and 27% of visits occurred on or after 2015, the year of idebenone approval for LHON in the EU. Idebenone use was recorded for 113 patients post baseline visit, after which they were no longer considered treatment-naïve; in these patients, data obtained from and after first idebenone use were excluded from further analysis.

The majority (175/219; 79.9%) of patients were male (4:1 male-to-female ratio) and had first symptom onset between 15–35 years of age (118/219; 53.9%), with a median (Q1–Q3) age of 26.0 (18.0–41.0) years. The distribution of causative mtDNA variants was: 71.7% (157/219) m.11778G>A, 14.6% (32/219) m.14484T>C, and 13.7% (30/219) m.3460G>A (Table 1). Symptoms developed in both eyes simultaneously in 45.7% of patients (100/219), with a median (Q1–Q3) delay of 2.0 (1.0–4.0) months between eyes in patients who developed symptoms sequentially (Supplemental Figure S1). A total of 36 eyes were excluded from further analyses due to the following reasons: unknown month of symptom onset (n=1); symptoms never developed in the second eye (n=8); patient started taking idebenone before symptom onset in the second eye (n=4), and no post-baseline assessment (n=23). A total of 402 eyes were available for analysis with a median (Q1–Q3) observation time of 7.2 (2.2–37.4) months (mean [range]; 29.7 [0.1–196.4]). Of these, 107 and 46 eyes had an available 12- and/or 24-month assessment, respectively (Table S2). At baseline, 4.7% (19/402) of eyes were in the chronic phase; due to this small number, only eyes that were subacute/dynamic at baseline are described in the following results. Demographics and baseline characteristics among the mtDNA variants were comparable (Table 2). By age at first symptom onset, most eyes from patients aged <15 years belonged to males (47/51; 92.2%); however, this proportion decreased with age and 59.6% (28/47) of eyes in the >50 years group belonged to males.

**Table 1:** Demographics and baseline characteristics of all patients by mtDNA variant (N=219)

Characteristic	Overall N=219	m.11778G>A n=157	m.3460G>A n=30	m.14484T>C n=32
*Sex, n (%)*				
Female	44 (20.1)	29 (18.5)	6 (20.0)	9 (28.1)
Male	175 (79.9)	128 (81.5)	24 (80.0)	23 (71.9)
*Age at first symptom onset, years*				
Mean (± SD)	29.8 (15.0)	29.9 (14.9)	28.1 (14.8)	31.1 (16.1)
Median (Q1–Q3)	26.0 (18.0–41.0)	26.0 (18.0–39.0)	23.0 (17.0–39.0)	26.5 (17.5–45.5)
Min–Max	6.0–68.0	6.0–68.0	7.0–65.0	8.0–63.0
*Age at baseline, years*				
Mean (± SD)	30.0 (15.0)	30.1 (14.9)	28.5 (14.7)	31.3 (16.1)
Median (Q1–Q3)	26.0 (18.0–41.0)	26.0 (18.0–39.0)	23.0 (17.0–40.0)	27.0 (17.5–45.5)
Min–Max	6.0–68.0	6.0–68.0	8.0–65.0	9.0–63.0
*Time since first symptom onset at baseline, n (%)*				
≤1 year	209 (95.4)	149 (94.9)	29 (96.7)	31 (96.9)
>1 year	10 (4.6)	8 (5.1)	1(3.3)	1 (3.1)
*Time since first symptom onset at baseline, months*				
Mean (± SD)	3.4 (5.6)	3.3 (5.2)	3.4 (8.6)	3.8 (3.2)
Median (Q1–Q3)	1.7 (0.7–3.9)	1.7 (0.7–3.7)	1.3 (0.8–3.1)	3.1 (1.1–6.1)
Min–Max	0.0–48.1	0.0–40.0	0.0–48.1	0.0–12.8
*Eyes affected at baseline per patient, n (%)*				
1 eye	64 (29.2)	52 (33.1)	5 (16.7)	7 (21.9)
2 eyes	155 (70.8)	105 (66.9)	25 (83.3)	25 (78.1)
*Difference in symptom onset between eyes, months*				
Mean (± SD)	2.9 (2.4)	2.9 (2.4)	2.6 (1.5)	3.0 (3.4)
Median (Q1–Q3)	2.0 (1.0–4.0)	2.0 (1.0–4.0)	2.6 (1.0–3.9)	1.5 (1.0–4.0)
Min–Max	0.1–12.0	0.3–12.0	0.1–5.0	0.2–11.2

*mtDNA* mitochondrial DNA, *Q1–Q3* interquartile range, *SD* standard deviation

**Table 2:** Demographics and baseline characteristics of eyes subacute/dynamic at baseline (n=383), by mtDNA variant and age at first symptom onset

Characteristic	Overall n=383	m.11778G>A n=273	m.3460G>A n=55	m.14484T>C n=55	<15 years n=51	15–35 years n=197	>35 years^a n=135^	>50 years^a n=47^
*Variant, n (%)*								
m.11778G>A	273 (71.3)	–	–	–	33 (64.7)	147 (74.6)	93 (68.9)	36 (76.6)
m.3460G>A	55 (14.4)	–	–	–	8 (15.7)	27 (13.7)	20 (14.8)	4 (8.5)
m.14484T>C	55 (14.4)	–	–	–	10 (19.6)	23 (11.7)	22 (16.3)	7 (14.9)
*Age at baseline, years*								
Mean (± SD)	30.0 (15.2)	30.3 (15.2)	28.8 (15.1)	30.2 (15.8)	10.9 (2.6)	22.8 (5.5)	47.9 (8.7)	58.2 (4.5)
Median (Q1–Q3)	26.0 (18.0–41.0)	26.0 (18.0–40.0)	22.0 (17.0–43.0)	25.0 (17.0–45.0)	10.0 (9.0–13.0)	22.0 (18.0–27.0)	46.0 (40.0–55.0)	57.0 (55.0–62.0)
Min–Max	6.0–68.0	6.0–68.0	8.0–65.0	9.0–63.0	6.0–15.0	15.0–36.0	36.0–68.0	52.0–68.0
*Sex, n (%)*								
Female	79 (20.6)	51 (18.7)	11 (20.0)	17 (30.9)	4 (7.8)	28 (14.2)	47 (34.8)	19 (40.4)
Male	304 (79.4)	222 (81.3)	44 (80.0)	38 (69.1)	47 (92.2)	169 (85.8)	88 (65.2)	28 (59.6)
*Time since first symptom onset at baseline, months*								
Mean (± SD)	2.2 (2.3)	2.1 (2.2)	1.5 (1.3)	3.4 (2.7)	2.4 (2.5)	2.0 (2.1)	2.3 (2.3)	2.2 (2.3)
Median (Q1–Q3)	1.3 (0.6–3.3)	1.3 (0.5–3.1)	1.0 (0.7–2.1)	2.6 (1.0–1.6)	2.0 (0.3–3.5)	1.2 (0.7–2.7)	1.3 (0.6–3.6)	1.3 (0.5–3.3)
Min–Max	0.0–10.8	0.0–10.8	0.0–4.8	0.0–9.9	0.0–10.7	0.0–10.8	0.0 (10.8)	0.0–6.8
*Time in observation, months*								
Mean (± SD)	30.0 (45.8)	31.9 (45.3)	21.3 (35.0)	29.2 (56.4)	48.0 (55.2)	31.6 (49.3)	20.8 (32.7)	18.5 (26.8)
Median (Q1–Q3)	6.9 (2.1–37.4)	7.8 (2.7–43.9)	4.6 (1.1–26.4)	2.5 (1.6–19.3)	22.0 (1.7–91.1)	6.4 (2.1–42.3)	6.9 (3.0–23.8)	7.2 (3.0–23.8)
Min–Max	0.1–196.4	0.1–187.5	0.2–161.8	0.2–196.4	0.3–168.0	0.1–196.4	0.2–130.3	0.4–121.3

^a^Eyes could fall into more than one age group, therefore the percentages do not add up to 100. *mtDNA* mitochondrial DNA, *Q1–Q3* interquartile range, *SD* standard deviation

The male-to-female ratio of eyes for each variant was 4.4:1, 4.0:1 and 2.2:1 for m.11778G>A, m.3460G>A, and m.14484T>C, respectively (Table S3). The distribution of each variant differed slightly by sex, with a slightly higher proportion of m.14484T>C (21.5%) and a lower proportion of m.11778G>A (64.6%) in eyes belonging to females compared with males (12.5% and 73.0%, respectively). Eyes from female patients were associated with older age at both first symptom onset and at baseline than eyes from males (median age at first symptom onset and at baseline: 40.0 years [females] vs 23.0 years [males]). Mean time since symptom onset was identical between sexes (2.2 months), indicating comparable disease stage at baseline despite slightly greater variability in eyes from females (Table S3).

### Clinically relevant outcomes

**Fig. 1:**
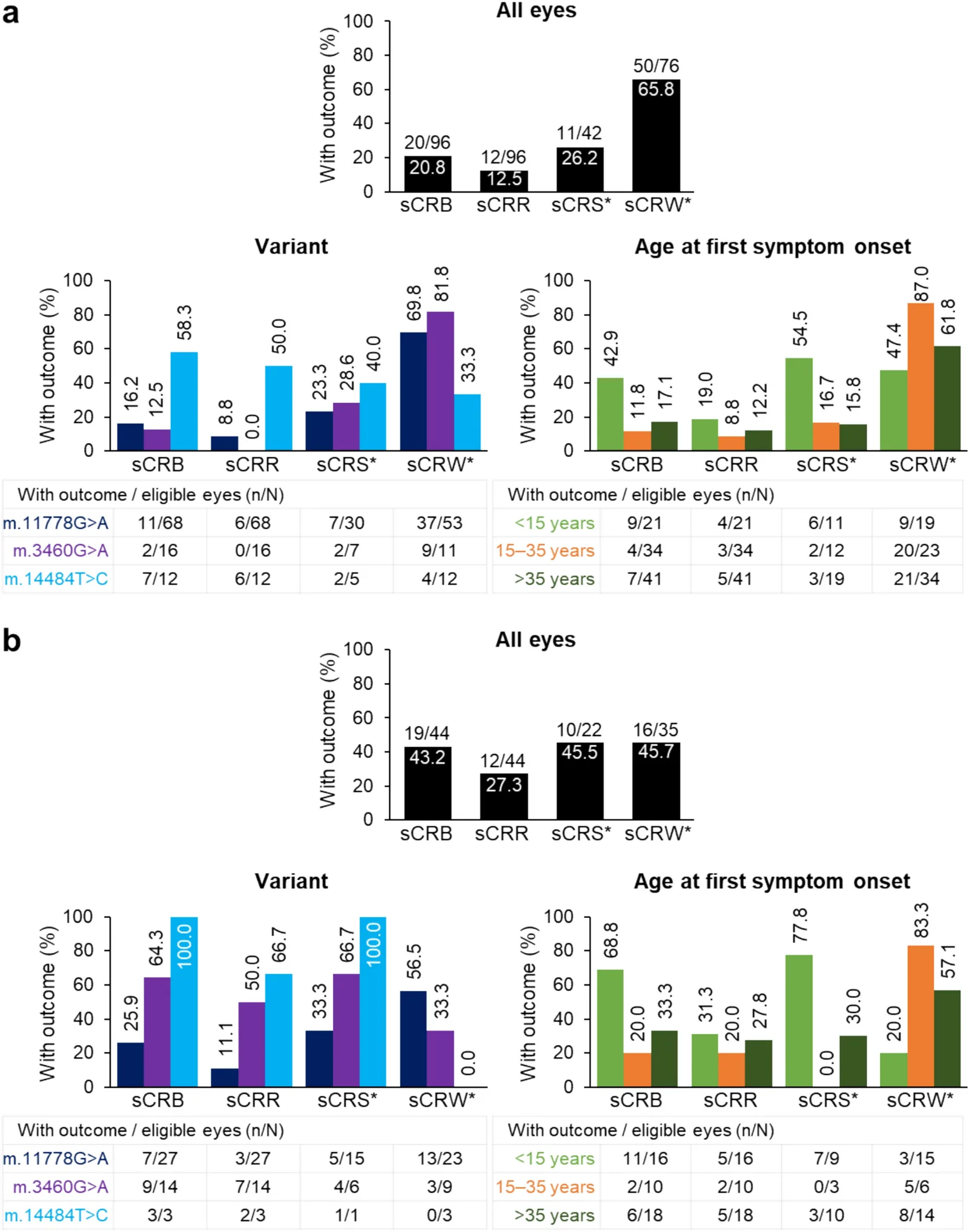
Clinical outcome rates in eyes subacute/dynamic at baseline for all eyes with an available VA assessment, by causative mtDNA variant, and by age at first symptom onset in the **a** 12-month and **b** 24-month analyses. *Only those eyes eligible for these definitions at baseline were used to calculate the proportion. *mtDNA* mitochondrial DNA, *sCRB* spontaneous clinically relevant benefit, *sCRR* spontaneous clinically relevant recovery, *sCRS* spontaneous clinically relevant stabilization, *sCRW* spontaneous clinically relevant worsening, *VA* visual acuity

Analysis of subacute/dynamic eyes for the primary endpoint showed 20.8% (20/96) experienced sCRB at 12 months post-baseline, which was due to 12 eyes achieving sCRR and 11 eyes sCRS (eyes with both an sCRR and sCRS were counted once) (Fig. 1A). Of the 44 eyes included in the 24-month analysis, 43.2% (19/44) experienced sCRB (Fig. 1B).

Eyes with the m.14484T>C variant showed the best outcomes in both the 12- and 24-month analyses, although the number of eyes was small, with the highest proportion experiencing sCRR (50.0% [6/12] and 66.7% [2/3], respectively), and the lowest proportion experiencing sCRW (33.3% [4/12] and 0.0% [0/3], respectively). In the 12-month analysis, eyes with the m.3460G>A variant showed the worst outcomes among the variants, with the lowest proportion experiencing sCRR (0% [0/16]) and the highest proportion experiencing sCRW (81.8% [9/11]), whereas in the 24-month analysis, eyes with the m.11778G>A variant had the worst outcomes (sCRR 11.1% [3/27] and sCRW 56.5% [13/23]).

Affected eyes of patients aged <15 years at first symptom onset had the best outcomes among the age groups, with the highest proportion experiencing sCRR in both the 12- and 24-month analyses (19.0% [4/21] and 31.3% [5/16], respectively) and the lowest proportion with sCRW (47.4% [9/19] and 20.0% [3/12], respectively). Affected eyes of patients aged >35 years at first symptom onset showed slightly better outcomes than the 15–35 years group, with a higher proportion experiencing sCRR in both analyses (12.2% vs 8.8% [12-month analysis] and 27.8% vs 20.0% [24-month analysis], respectively). Although eyes in these two groups still showed high rates of sCRW, those aged >35 years at first symptom onset had a lower likelihood of experiencing sCRW compared with the 15–35 years group in both analyses (61.8% vs 87.0% [12-month analysis] and 57.1% vs 83.3% [24-month analysis] in the >35 years and 15–35 years groups, respectively).

Analysis by sex revealed slightly worse outcomes for eyes from females versus males (Supplemental Figure S2). None of the eyes from females experienced sCRS in either analysis, and a higher proportion experienced sCRW in both analyses compared with eyes from males (69.2% vs 65.1% [12-month analysis] and 83.3% vs 37.9% [24-month analysis]).

**Fig. 2:**
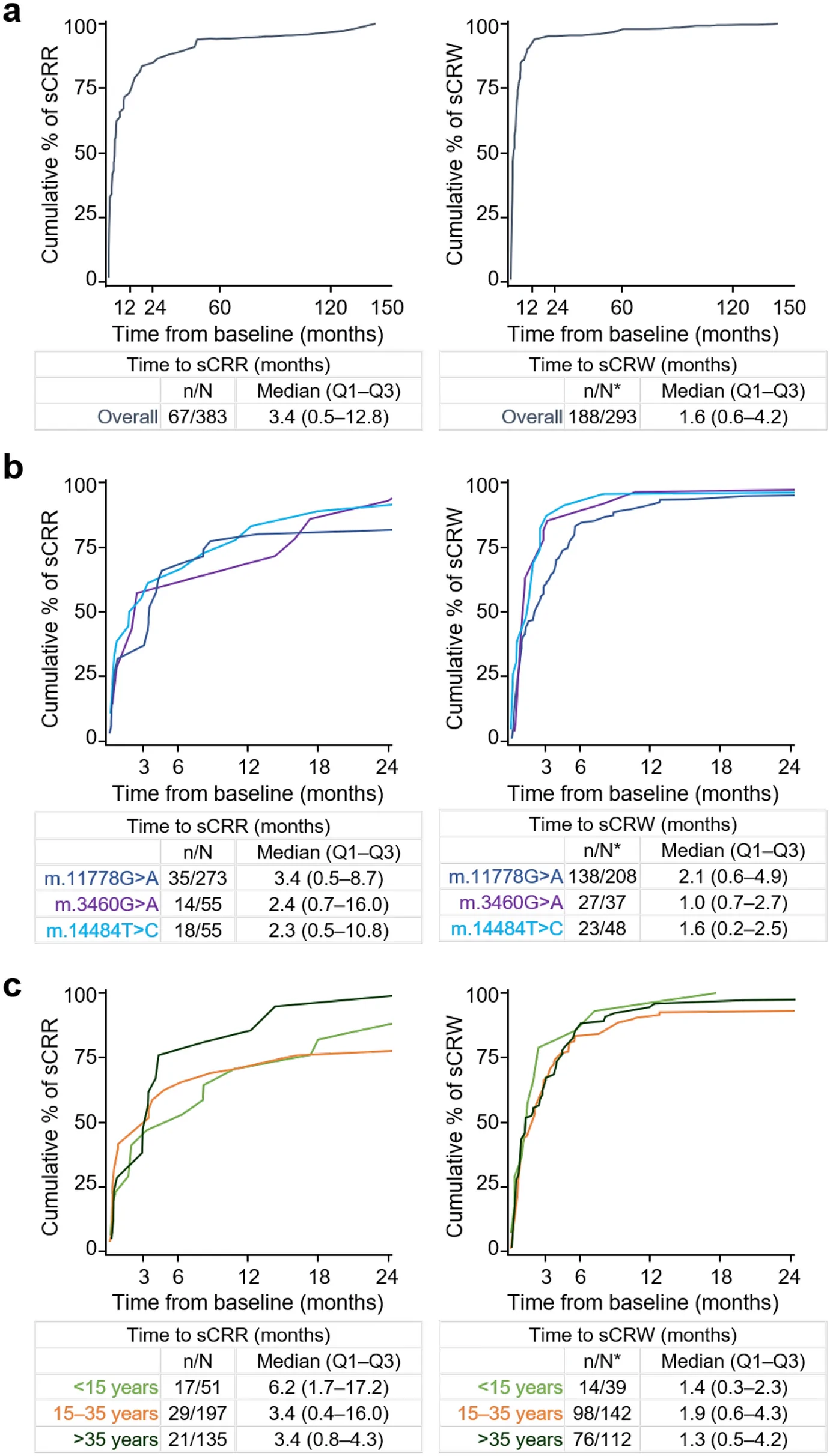
Cumulative proportion curves of eyes subacute/dynamic at baseline that showed sCRR (n=67) or sCRW (n=188) at their last assessment. Curves are shown up to 150 months for **a** all subacute/dynamic eyes, and up to 24 months by **b** causative mtDNA variant and **c** age at first symptom onset. *Only those eyes eligible for sCRW (i.e., those eyes on-chart at baseline) are shown in the denominator. *mtDNA* mitochondrial DNA, *sCRR* spontaneous clinically relevant recovery, *sCRW* spontaneous clinically relevant worsening, *Q1–Q3* interquartile range

Overall (Fig. 2A), 67 eyes showed sCRR at their last assessment, occurring at a median time of 3.4 months from baseline. Notably, a quarter of these eyes did so ≥12.8 months after their baseline visit. Substantially more eyes (n=188) showed sCRW at their last assessment, occurring at a much shorter median time of 1.6 months from baseline. Median time to sCRR and sCRW did not differ greatly among variants (Fig. 2B); however, a quarter of m.3460G>A eyes with sCRR at last assessment reached this milestone ≥16.0 months after baseline. Affected eyes of patients aged <15 years at first symptom onset had a longer median time to sCRR of 6.2 months compared with the other age groups (Fig. 2C). Although eyes of patients aged 15–35 years and >35 years at first symptom onset had the same median time to sCRR, 75% of eyes in patients aged >35 years experienced sCRR before 4.3 months post-baseline. All age groups had similar median times to sCRW. By sex (Supplemental Figure S3), eyes from male and female patients experienced sCRR and sCRW at roughly similar times.

Post hoc application of a ≥15-letter (±0.3 logMAR) threshold for spontaneous clinically relevant outcomes resulted in little to no change in observed outcome rates (Supplemental Figure S4).

### Changes in visual acuity

Changes in VA from baseline in the 12- and 24-month analyses are shown in Table 3. Of the three variants, m.11778G>A eyes showed the largest median (Q1–Q3) VA deterioration (0.50 [0.00–0.86] logMAR) from baseline to 12-months, followed by m.3460G>A (0.31 [0.00–0.50] logMAR), whereas m.14484T>C eyes demonstrated improvement (−0.24 [−0.78–0.46] logMAR). Similar relative differences among the variants could be seen in the 24-month analysis except for the improvement with m.3460G>A. Regression analysis (Table 4) showed that the VA change in m.14484T>C eyes was significantly more favorable compared with the reference m.11778G>A group in the 12- and 24-month analyses (p=0.0001 and p=0.0006, respectively). VA change in m.3460G>A eyes was significantly more favorable only in the 24-month analysis (p=0.0064) versus m.11778G>A eyes.

**Table 3:** VA of eyes subacute/dynamic at baseline in the 12- and 24-month analyses, by mtDNA variant and age at first symptom onset

	Overall	m.11778G>A	m.3460G>A	m.14484T>C	<15 years	15–35 years	>35 years^a^	>50 years^a^
*12-month analysis*								
	n=96	n=68	n=16	n=12	n=21	n=34	n=41	n=16
*Baseline VA (logMAR)*								
Mean (± SD)	0.96 (0.58)	0.95 (0.59)	1.06 (0.67)	0.95 (0.40)	0.76 (0.50)	1.12 (0.64)	0.94 (0.54)	0.81 (0.55)
Median (Q1–Q3)	1.00 (0.45–1.40)	1.00 (0.40–1.40)	1.30 (0.40–1.80)	1.00 (0.78–1.30)	0.70 (0.40–1.00)	1.30 (0.70–1.80)	1.00 (0.40–1.30)	0.80 (0.30–1.19)
Min–Max	0.00–1.80	0.00–1.80	0.10–1.80	0.10–1.54	0.00–1.80	0.00–1.80	0.00–1.80	0.00–1.80
*12-month VA (logMAR)*								
Mean (± SD)	1.35 (0.56)	1.42 (0.50)	1.47 (0.58)	0.79 (0.53)	0.94 (0.51)	1.57 (0.44)	1.38 (0.56)	1.41 (0.63)
Median (Q1–Q3)	1.64 (1.00–1.80)	1.80 (1.00–1.80)	1.80 (1.30–1.80)	0.61 (0.49–1.35)	1.00 (0.52–1.10)	1.80 (1.54–1.80)	1.80 (1.10–1.80)	1.80 (1.10–1.80)
Min–Max	0.00–1.80	0.00–1.80	0.15–1.80	0.10–1.54	0.00–1.80	0.15–1.80	0.10–1.80	0.15–1.80
*Magnitude of VA change from baseline (logMAR)*								
Mean (± SD)	0.39 (0.67)	0.48 (0.65)	0.42 (0.58)	−0.16 (0.67)	0.18 (0.51)	0.46 (0.68)	0.44 (0.72)	0.60 (0.80)
Median (Q1–Q3)	0.31 (0.00–0.80)	0.50 (0.00–0.86)	0.31 (0.00–0.50)	−0.24 (−0.78–0.46)	0.00 (−0.10–0.49)	0.45 (0.00–0.82)	0.32 (0.00–0.90)	0.66 (−0.04–1.29)
Min–Max	−1.30–1.80	−1.30–1.80	−0.15–1.60	−0.90–1.02	−0.78–1.12	−1.30–1.80	−0.90–1.80	−0.78–1.80
*24-month analysis*								
	n=44	n=27	n=14	n=3	n=16	n=10	n=18	n=9
*Baseline VA (logMAR)*								
Mean (± SD)	0.95 (0.57)	0.81 (0.54)	1.17 (0.62)	1.13 (0.30)	0.76 (0.47)	1.29 (0.49)	0.92 (0.63)	0.77 (0.56)
Median (Q1–Q3)	0.89 (0.40–1.39)	0.70 (0.30–1.00)	1.39 (0.60–1.80)	1.30 (0.78–1.30)	0.65 (0.40–1.00)	1.30 (0.70–1.80)	0.74 (0.30–1.48)	0.62 (0.30–1.30)
Min–Max	0.00–1.80	0.00–1.80	0.22–1.80	0.78–1.30	0.00–1.80	0.70–1.80	0.20–1.80	0.22–1.80
*24-month VA (logMAR)*								
Mean (± SD)	1.10 (0.65)	1.29 (0.60)	0.93 (0.61)	0.22 (0.33)	0.76 (0.41)	1.39 (0.59)	1.25 (0.74)	0.97 (0.92)
Median (Q1–Q3)	1.20 (0.60–1.80)	1.52 (0.70–1.80)	1.00 (0.52–1.30)	0.05 (0.00–0.60)	0.79 (0.46–1.00)	1.66 (1.00–1.80)	1.66 (0.60–1.80)	1.52 (0.00–1.80)
Min–Max	0.00–1.80	0.00–1.80	0.00–1.80	0.00–0.60	0.00–1.52	0.00–1.80	0.00–1.80	0.00–1.80
*Magnitude of VA change from baseline (logMAR)*								
Mean (± SD)	0.16 (0.74)	0.48 (0.67)	−0.24 (0.44)	−0.91 (0.64)	0.00 (0.50)	0.10 (0.65)	0.33 (0.95)	0.20 (1.08)
Median (Q1–Q3)	0.00 (−0.26–0.55)	0.18 (0.00–1.12)	−0.20 (−0.50–0.00)	−1.26 (−1.30– −0.18)	0.00 (−0.35–0.14)	0.11 (0.00–0.30)	0.00 (−0.22–1.40)	0.00 (−0.30–1.18)
Min–Max	−1.30–1.60	−0.48–1.60	−1.30–0.30	−1.30– −0.18	−0.70–1.12	−1.30–1.10	−1.30–1.60	−1.30–1.50

^a^Some eyes overlap between age groups. *logMAR* logarithm of the minimum angle of resolution, *mtDNA* mitochondrial DNA, *Q1–Q3* interquartile range, *SD* standard deviation, *VA* visual acuity

**Table 4:** Linear regressions of patient and disease variables for change in VA in the 12- and 24-month analyses of eyes subacute/dynamic at baseline

	12-month analysis (n=96)		24-month analysis (n=44)	
Variable	β Coefficient [95% CI]	p-value	β Coefficient [95% CI]	p-value
Baseline VA	−0.70 [−0.88,−0.53]	<0.0001	−0.53 [−0.84,−0.23]	0.0011
*Variant*				
m.11778G>A (reference group)				
m.3460G>A	0.02 [−0.25,0.29]	0.8860	−0.53 [−0.91,−0.16]	0.0064
m.14484T>C	−0.63 [−0.94,−0.32]	0.0001	−1.23 [−1.90,−0.56]	0.0006
Baseline VA	−0.78 [−0.96,−0.60]	<0.0001	−0.82 [−1.17,−0.47	<0.0001
*Age at first symptom onset*				
15–35 years (reference group)				
<15 years	−0.55 [−0.83,−0.27]	0.0002	−0.54 [−1.06,−0.01]	0.0463
>35 years	−0.16 [−0.39,0.07]	0.1730	−0.08 [−0.57,0.42]	0.7612
Baseline VA	−0.70 [−0.89,−0.51]	<0.0001	−0.71 [−1.04,−0.39]	0.0001
*Sex*				
Male (reference group)				
Female	0.02 [−0.28,0.32]	0.9079	0.55 [0.07,1.02]	0.0251

*CI* confidence interval, *VA* visual acuity

When looking at eyes by age at first symptom onset (Table 3), those from patients aged 15–35 years experienced the most extensive worsening in VA among the age groups in both the 12- and 24-month analyses (median [Q1–Q3]; 0.45 [0.00–0.82] and 0.11 [0.00–0.30] logMAR, respectively), whereas the <15 years age group showed the least change (median [Q1–Q3]; 0.00 [−0.10–0.49] and 0.00 [−0.35–0.14] logMAR, respectively). Regression analysis (Table 4) revealed that the VA change in eyes of patients aged <15 years at first symptom onset was significantly more favorable compared with the reference (15–35 years group) in the 12- and 24-month analyses (p=0.0002 and p=0.0463, respectively). Although eyes of patients aged >35 years at first symptom onset tended to have a less positive median change in VA (i.e., less VA worsening) in both the 12- and 24-month analyses versus the 15–35 years group, regression analysis did not show a significant difference in these two age groups.

Median change in VA was generally worse in eyes from female patients versus male patients in both the 12- and 24-month analyses (Table S4). Despite the small numbers of eyes from female patients, the median change in VA from baseline was typically worse than the equivalent age group in males in both analyses. In the regression analysis (Table 4), the difference between eyes from male and female patients was only significant at the 24-month analysis (p=0.0251).

**Fig. 3:**
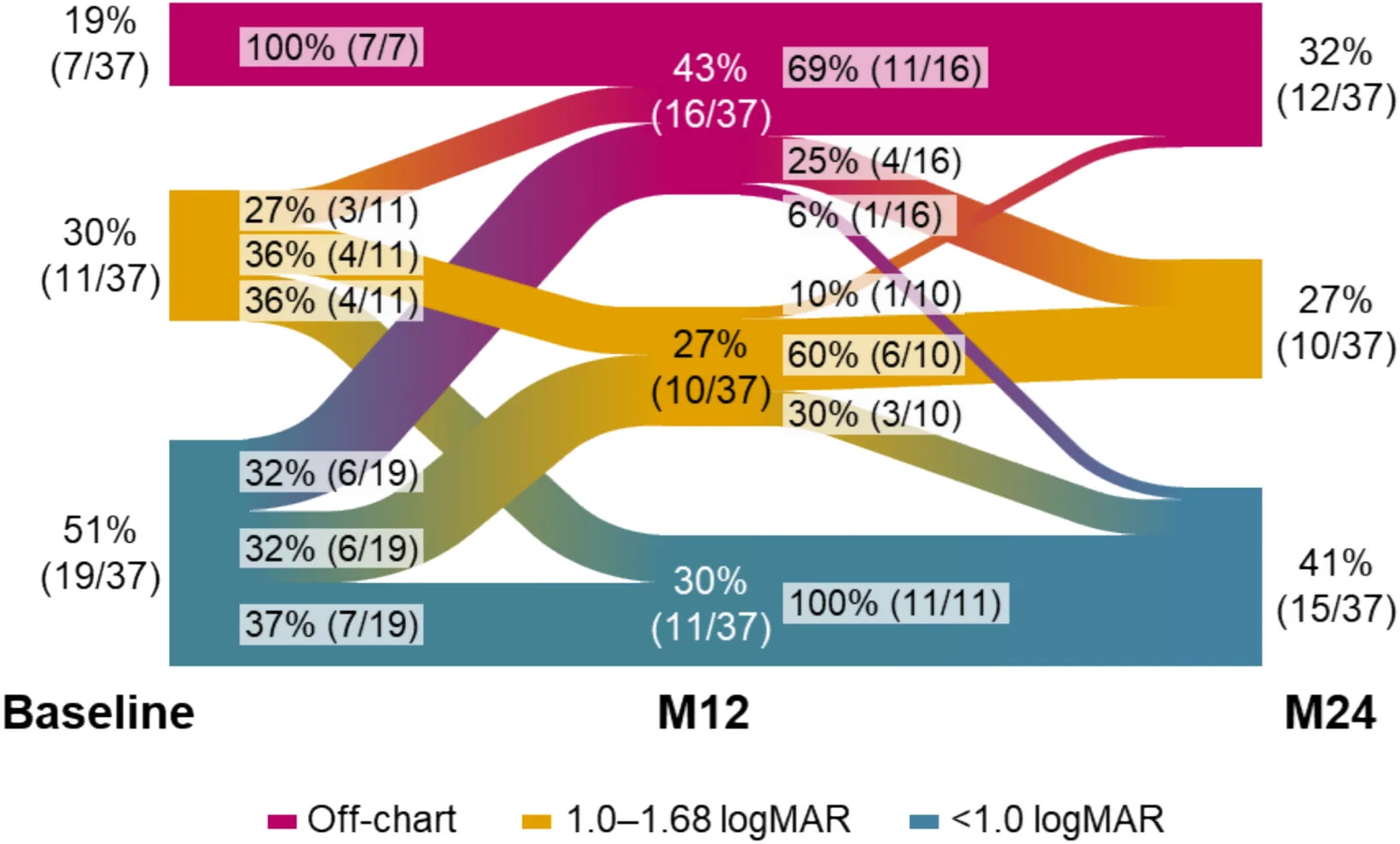
Transitions between VA blindness categories over time. Figure shows all eyes subacute/dynamic at baseline that were included in both the 12- and 24-month analyses (n=37). Cross-overs illustrate when eyes in particular categories transition to another category. Percentages reflect the proportion of eyes in that category transitioning to connected category at the following timepoint. *logMAR* logarithm of the minimum angle of resolution, *M* month, *VA* visual acuity

In all models, baseline VA showed a significant contribution to change in VA (Table S5). Baseline VA had negative coefficients in all regressions, indicating that a worse VA at baseline was correlated with a larger improvement in VA from baseline (Table 4). Longitudinal comparison of outcomes from the 12- and 24-month analyses was not possible due to the different populations of eyes; however, data were available for 37 eyes with both 12- and 24-month VA assessments (Fig. 3). Changes in VA blindness category showed that the overall proportion of eyes declined in the <1.0 logMAR category and increased in the off-chart category, demonstrating a general decline in VA. Although eyes transitioned into both better and worse blindness categories in both time periods, nearly a quarter (24% [9/37]) of eyes changed blindness categories between their 12- and 24-month VA assessments.

**Fig. 4:**
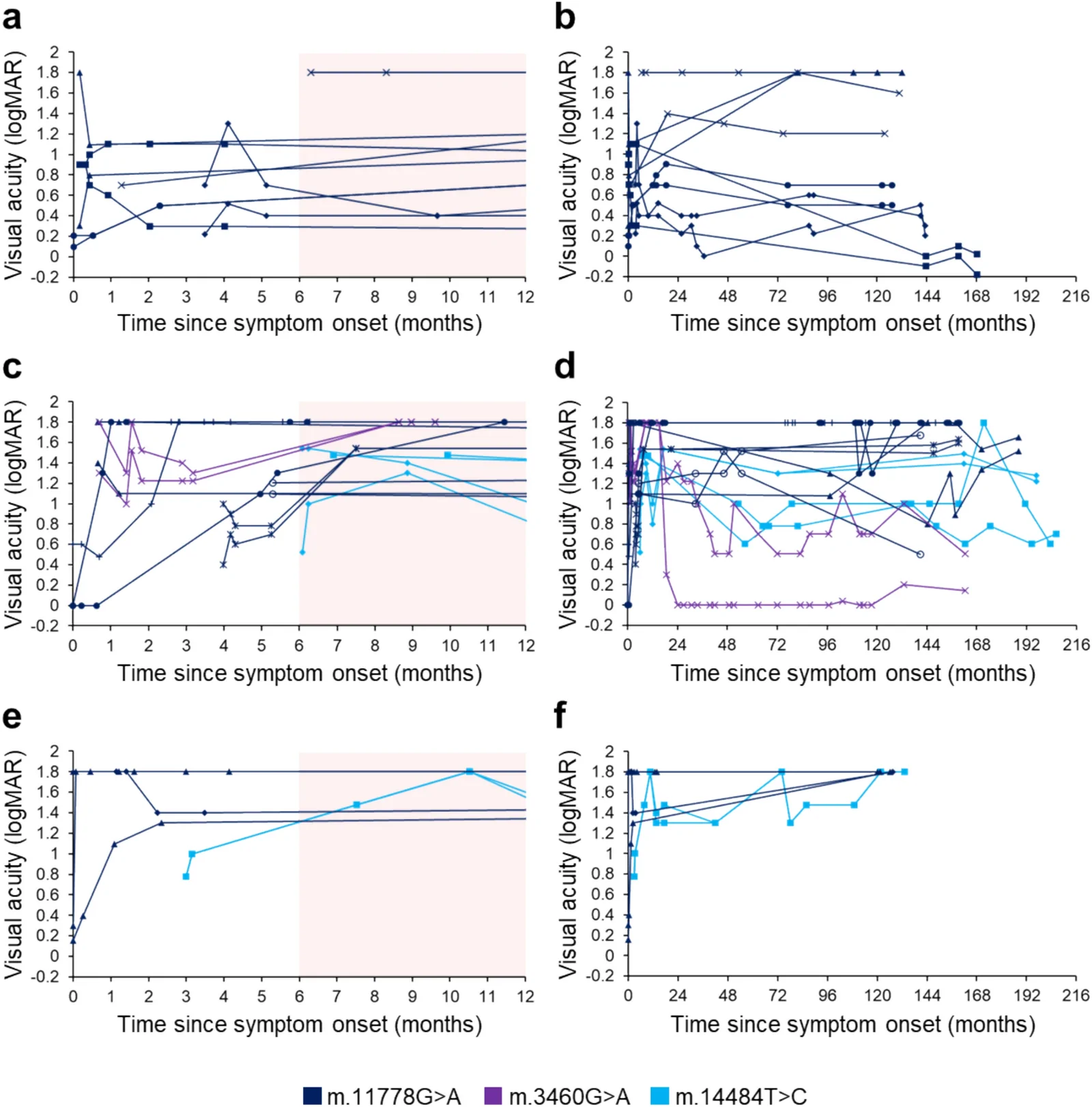
VA by time since symptom onset in eyes subacute/dynamic at baseline with more than 10 years of follow-up at last visit (n=32), in those aged **a, b** <15 years, **c, d** 15–35 years and **e, f** >35 years at first symptom onset. Panels (**a, c, e**) show the first 12 months since symptom onset, with red shading indicating when the eye is considered to be in the dynamic disease stage. Panels (**b, d, f**) show the entire observation period. Different markers within a graph represent eyes belonging to different patients. Each patient has two lines representing the VA trajectory of the left and right eye. *logMAR* logarithm of the minimum angle of resolution, *VA* visual acuity

VA trajectories for the 32 eyes that were subacute/dynamic at baseline and had a follow-up duration of more than 10 years showed both inter- and intrapatient variability, with many eyes showing variable rates of decline or changes in trajectory direction (Fig. 4). Notably, and in all variant and age subgroups, nadir did not always occur within the first year of symptom onset, and changes in VA occurred well into the chronic stage in many eyes.

## Discussion

The CRS-2 study offers the largest contemporary dataset on the natural history of LHON VA in idebenone treatment-naïve eyes. In this European cohort of patients, baseline demographics were largely similar to previous reports and the known epidemiology of LHON; consisting of a male majority (80%), mean age of onset of 30 years and with m.11778G>A as the predominant mtDNA pathogenic variant (72%) [10]. The large population size of CRS-2 facilitated a comprehensive analysis of subgroup demographics, with several findings consistent with those of a large, predominantly North American survey of 1,517 participants affected by LHON [11]. This survey demonstrated a 3:1 male-to-female ratio, which the authors highlighted was lower than the traditionally reported ratio of 5:1 [11]. In CRS-2, the male-to-female ratio lay roughly in between these two extremes, at 4:1.

Age at first symptom onset did not largely differ among the three mtDNA variants. Occurrence of the m.14484T>C variant was proportionally higher in eyes from female patients compared with eyes from male patients, and m.11778G>A was proportionally lower. This contrasts with previous observations in which the distribution was similar for both sexes (approximately two-thirds with m.11778G>A, with the remaining third split roughly equally between m.14484T>C and m.3460G>A) [11].

The proportion of eyes from male patients decreased with increasing age at first symptom onset, with a roughly 1.5:1 male-to-female ratio in the >50 years age category, nearing the reported 1:1 ratio in patients aged >45 years in the predominantly North American survey [11]. The authors of this survey noted that 10% of their participants experienced LHON symptom onset after 50 years of age, nearly double that of previous reports [3, 11, 35]; the CRS-2 cohort had a similar proportion, with 12.3% of eyes from patients aged >50 years at first symptom onset.

The CRS-2 study was designed to provide a contemporary, external control group of idebenone treatment-naïve patients to the LEROS study population and included similar endpoints and analysis timepoints [24]. The subsets of CRS-2 eyes with similar follow-up times (i.e., either 12±3 or 24±3 months) facilitated subgroup comparisons of outcomes that would be otherwise challenging to perform in a population with varying follow-up frequencies and durations.

Predictors of relative visual outcomes were consistent with previous reports. Among the three pathogenic variants, eyes with the m.14484T>C variant showed the best outcomes, with rates of sCRR (50.0% and 66.7% in the 12- and 24-month analyses, respectively) consistent with previously reported rates of 37–71% [34, 36]. Interestingly, eyes with the m.14484T>C variant had a longer time since first symptom onset to their baseline visit compared with the other two variants, as well as a higher proportion of patients for which both eyes were affected at first symptom onset, which may potentially be linked to the milder progression of the disease.

Eyes with the m.11778G>A variant are reported to have the worst prognosis, with spontaneous visual recovery rates of 4–25%, whereas those with the m.3460G>A variant have an intermediate prognosis, with recovery rates of 15–25% [5, 28, 34, 36–38]. In the present study, m.11778G>A eyes showed sCRR rates of 8.8% and 11.1%, whereas eyes with the m.3460G>A variant showed rates of 0% and 50.0% in the 12- and 24-month analyses, respectively. Interestingly, data from regression analysis showed that VA change in m.3460G>A eyes was significantly more favorable than in those with the m.11778G>A variant, but only in the 24-month analysis. This, in combination with a median VA change of −0.20 logMAR from baseline in the 24-month analysis, suggests increased spontaneous VA improvement in m.3460G>A eyes (relative to m.11778G>A) over a longer time course. Although these highly varying rates may be partially attributable to the small sample size, it is interesting to note that the distribution of time to sCRR was much wider in eyes with the m.3460G>A variant when considering all eyes, regardless of timepoint. Together, these observations highlight the importance of considering time since onset when evaluating recovery rates and raise the question of whether different variants show different temporal patterns of spontaneous VA recovery.

Eyes from patients who were younger at first symptom onset tended to show the best outcomes among the age groups, consistent with reports describing better visual prognosis in patients with childhood-onset versus adult-onset LHON [2, 28, 36, 59]. Interestingly, time to sCRR and sCRW differed in eyes aged <15 years at first symptom onset compared with the two older age groups. In eyes that experienced sCRW, 75% did so within 2.3 months from baseline, and in those experiencing sCRR, the distribution of time to sCRR skewed much later compared with the two older age groups. Although eyes in the >35 years group tended to have better VA at baseline than the 15–35 years group in both the 12- and 24-month analyses, the two groups did not differ greatly with regards to clinically relevant outcomes or changes in VA. Although the CRS-2 study did not collect information on external factors, familial case series data from Carelli et al. draw attention to the potential impact of environmental factors, such as smoking or alcohol consumption, in driving later onset of disease [40]. It may be that the later disease onset observed in CRS-2 is a result of similar environmental triggers.

The classic view of LHON disease progression describes a rapid decline in VA, often reaching a nadir in 4 to 6 months, followed by relative stabilization of poor VA roughly one year after symptom onset [1]. This understanding forms the basis of several clinical study design parameters such as the timing of endpoints and outcome measures. Retrospective extraction of all idebenone treatment-naïve VA assessments in a large group of patients offered the valuable opportunity to visualize disease progression in a more granular way and to identify potential patterns, outliers and presence of subgroups that may be otherwise obscured if summarized. Unsurprisingly, the CRS-2 dataset revealed substantial inter- and intrapatient heterogeneity in VA trajectories, highlighting potential drawbacks of summarizing these into a single curve. Substantial shifts in VA leading to a change in blindness category could be observed between 12 and 24 months after baseline in many cases that had both available assessments. In those with less regular assessments, spontaneous changes in VA, including recovery, could be observed well into the chronic stage and even years after symptom onset.

These observations were consistent with previous reports visualizing LHON natural history [30, 41]. Indeed, the recently published REALITY study, including 23 patients aged ≥15 years at onset and with the m.11778G>A variant, showed a lack of VA stabilization even after 2 years, with a decline in VA observed up to 150 months from disease onset [30]. A more extensive model on VA progression in m.11778G>A eyes, which consolidated data from the above study and from a systematic literature review, presented overall improvement in VA from 12 to 30 months post symptom development, followed by a general decline from 30 to 52 months [41]. It must be noted, however, that both models included patients receiving idebenone, complicating interpretation of these results given the significant improvement in VA reported in the LEROS trial [24]. Altogether, these observations of VA trajectories highlight the need for caution when interpreting results that rely on nadir or last visit VA values.

To form the LEROS natural history group, eyes from the CRS-2 study were pooled with those from CRS-1 [24, 32, 33], and were matched to idebenone-treated eyes using an algorithm taking into consideration time from symptom onset. In CRS-2, 96 eyes were subacute/dynamic at baseline and had an available 12-month VA assessment. These eyes had similar demographics and baseline characteristics to the 193 matched subacute/dynamic LEROS natural history eyes at the 12-month timepoint [24]. In CRS-2, 20.8% of subacute/dynamic eyes with a 12-month assessment experienced sCRB (primary endpoint), versus 20.7% in the LEROS natural history group [24]. Reinforcing the application of the algorithm implemented in the LEROS study [24], the remaining outcomes, as well as those in the 24-month analyses, were also largely comparable between the two populations, with the exception of m.3460G>A eyes.

Eyes with the m.3460G>A variant had lower rates of sCRR in CRS-2 versus the LEROS natural history group at 12 months (0% vs 41.2%) and less so at 24 months (50.0% vs 61.1%), suggesting eyes with better outcomes may have been more frequently represented in the LEROS natural history group [24]. This could have been due to eyes with better outcomes having more frequent follow-up and opportunity for matching, or eyes showing quick worsening or longer time to recovery being lost to follow-up, or a combination of both.

An understanding of the natural history of a disease is crucial for designing studies, guiding clinical decisions and ultimately improving patient outcomes; however, this remains a challenge to study in a rare disease with low patient numbers and in which an approved treatment is available. In such cases, a retrospective case record survey is arguably the most suitable approach, but it is not without its limitations. As would be expected, not all data that may influence the progression of VA were recorded. Thus, variables known to impact disease progression such as lifestyle habits (e.g., smoking or alcohol consumption), concomitant supplementation and medication, or measures of stress were not collected. In addition, although VA data were standardized through conversion to logMAR units and predefined outcome definitions, the retrospective nature meant that assessment details such as testing procedure, chart type, and testing distance were not prospectively harmonized across sites, which may have contributed to measurement variability. Changes in LHON referral pathways, genetic testing availability and diagnostic awareness over time could have introduced ascertainment bias, given the long inclusion period.

Variability of visit frequency and observation duration are perhaps the most important limitations of the study design to consider. Due to the retrospective and observational nature of the study, regularity of follow-up visits between patients and for each patient were not consistent, as these were determined by factors such as individualized patient management, as well as differences in institutional and international guidelines. Follow-up time in the CRS-2 study ranged from 0.1 to 196.4 months; cases with short follow-up may be due to reasons that could introduce bias (e.g., start of idebenone treatment in patients with better VA, or reduced inclination to continue clinic visits due to poor VA), or due to reasons that simply reflect the nature of the study design, such as development of symptoms close to the end of study enrollment. Cases with short follow-up were not excluded in order to avoid any introduction of survivorship bias through preferential inclusion of patients with more complete clinical records. Patients with longer follow-up duration may exhibit a bias towards those with better or more fluctuating VA. Although tempting, rates observed between the 12- and 24-month analyses in this study should not be compared or seen as longitudinal changes, as many eyes were only included in one of the two and may have been influenced by the biases described above.

To remain consistent with methods utilized in the LEROS study [24], any recorded off-chart VA assessment was assigned a value of 1.8 logMAR in the CRS-2 study. However, it must be kept in mind that approaches to measure off-chart VA vary among different studies and can impact the comparability of some results [21, 42]. In CRS-2, the extent of both VA improvement and worsening between the on-chart range (up to a maximum of 1.68 logMAR) and the off-chart space would be underestimated compared to approaches that assign higher logMAR values for off-chart measures such as counting fingers, hand motion, and light perception. Further, in the CRS-2 study, any improvement or worsening within the off-chart space was not captured and thus, its magnitude not quantified as a gain or loss in logMAR.

Outcome definitions were also aligned with those used in the LEROS study. Although the Food and Drug Administration (FDA) has historically accepted changes of ≥15 letters (±0.3 logMAR) as clinically meaningful in retina therapy trials, the FDA has also acknowledged that a ≥10-letter change may be acceptable as a primary endpoint in certain contexts [43, 44]. In addition, post hoc application of the stricter 15-letter threshold had little impact on the rates of spontaneous clinically relevant outcomes observed in CRS-2, supporting the robustness of these results.

## Conclusions

Information on the natural history of LHON, and particularly in subgroups of interest, remains limited. The CRS-2 study collected demographic data and examined the natural history of LHON VA in a large population of idebenone treatment-naïve eyes. Despite the limitations faced using a retrospective study design, insight into changes in VA and relative differences among subgroups of interest were observed. These data further underscored the question of timing in disease progression, particularly the concept of a stabilized VA in the chronic phase of the disease. Future work could incorporate more sophisticated statistical analyses to examine trends within various subgroups. Assessment of optical coherence tomography-derived structural biomarkers and low-contrast VA in future studies may complement the functional findings presented in this study.

Overall, the CRS-2 study deepens our understanding of LHON disease progression, which could inform more targeted therapeutic approaches, improve study designs, and advance patient care. These findings characterize the time course and pattern of untreated disease progression, providing a benchmark against which therapeutic outcomes can be interpreted and future interventions evaluated.

## Supplementary information

The accompanying supplementary information contains the full list of secondary endpoints of the CRS-2 study and supplementary data including demographics and baseline characteristics, baseline VA, and change in VA for 12- and 24-month analyses for subacute/dynamic eyes. Below is the link to the electronic supplementary material.

Supplementary file1 (PDF 433 KB)

## Data Availability

The individual patient data reported in this study cannot be deposited in a public repository because these data are confidential medical records. To request access, please contact Thomas Klopstock (thomas.klopstock@med.uni-muenchen.de) for de-identified summary data.
