# Supplementary material for: Visual acuity changes in idebenone treatment‑naïve patients with Leber hereditary optic neuropathy: insights from the CRS-2 study
Source: J Neurol. 2026 Oct 28;273(11):690. doi: 10.1007/s00415-026-14181-6 (PMC13719768; doi:10.1007/s00415-026-14181-6)
Supplement: Supplementary file 1 [file 415_2026_14181_MOESM1_ESM.pdf]

SUPPLEMENTARY INFORMATION

Visual acuity changes in idebenone treatment-naïve patients with Leber hereditary optic neuropathy: Insights from the CRS-2 study - *Journal of Neurology*

Authors: Maciej R. Krawczynski, MD PhD, Judith van Everdingen, MD, Bart P. Leroy, MD PhD, Christophe Verny MD PhD, Nöel J.C. Bauer, MD PhD, Cécile Andris, MD, Birgit Lorenz MD PhD, Marcela Votruba, BMBCh PhD, Claudia Priglinger, MD, Neringa Jurkute, MD PhD, Xavier Llòria, MD, Magda Silva, Valerio Carelli, MD PhD, Patrick Yu-Wai-Man, MD PhD, Thomas Klopstock, MD

Corresponding author: Thomas Klopstock

Department of Neurology, Friedrich-Baur-Institute, LMU University Hospital, LMU Medizin, Ludwig-Maximilians-Universität (LMU) München, 80336 Munich, Germany; AND German Center for Neurodegenerative Diseases (DZNE), 81377 Munich, Germany

Email: Thomas.Klopstock@med.uni-muenchen.de

Appendix I. Full list of secondary endpoints.

In eyes that were subacute/dynamic at baseline:

- Proportion of eyes with sCRR at 12 months
- Proportion of eyes with sCRS at 12 months
- Proportion of patients with sCRB in at least one eye at 12 months
- Proportion of eyes/patients with off-chart VA at baseline in whom VA improves to better than or equal to 1.60 logMAR at 12 months
- Proportion of eyes/patients with VA in the categories of better than 1.0 logMAR, 1.0 to 1.68 logMAR, and above 1.68 logMAR at baseline, 12 months and 24 months
- Proportion of eyes with sCRB at 24 months

In eyes that were chronic at baseline:

- Proportion of eyes with sCRR at 12 months
- Proportion of eyes with sCRS at 12 months
- Proportion of eyes with sCRB at 12 months
- Proportion of patients with sCRB in at least one eye at 12 months
- Proportion of eyes/patients with off-chart VA at baseline in whom VA improves to better than or equal to 1.60 logMAR at 12 months
- Proportion of eyes/patients with VA in the categories of better than 1.0 logMAR, 1.0 to 1.68 logMAR, and above 1.68 logMAR at baseline, 12 months and 24 months
- Proportion of eyes with sCRB at 24 months

logMAR: logarithm of the minimum angle of resolution; sCRB: spontaneous clinically relevant benefit; sCRR: spontaneous clinically relevant recovery; sCRS: spontaneous clinically relevant stabilization; sCRW: spontaneous clinically relevant worsening; VA: visual acuity.

Table S1 List of IECs at each participating site that reviewed and approved the study protocol and all amendments.

Site number	Site	Name of PI	IEC
10101	C. H. U. Sart Tilman, Domaine Universitaire du Sart Tilman	Andris, Cécile	Comité d'Éthique Hospitalo-Facultaire Universitaire de Liège Centre Hospitalier Universitaire de Liège, Site du Sart Tilman
10102	Cliniques Universitaire Saint-Luc	Boschi, Antonella	Commission d'Ethique Biomédicale Hospitalo-Facultaire de l'UCL Cliniques Universitaires Saint-Luc
10103	Universitair Ziekenhuis Gent	Leroy, Bart	Ethisch Comité Universitair Ziekenhuis Gent
10104	CHU Saint-Pierre	Meunier, Audrey	Comité d'Éthique Centre Hospitalier Universitaire Saint-Pierre
10202	CHU Angers - Hôpital Hôtel Dieu	Verny, Christophe	CCTIRS Direction de la Recherche et de l'Innovation, Paris
10203	Centre Hospitalier National d'Ophtalmologie (CHNO) des Quinze-Vingts	Vignal, Catherine	CCTIRS Direction de la Recherche et de l'Innovation, Paris
10204	Hôpital Roger Salengro - CHU Lille	Defoort, Delemmes Sabine	CCTIRS Direction de la Recherche et de l'Innovation, Paris
10205	CHU Strasbourg - Hôpital Hautepierre	Dollfus, Hélène	CCTIRS Direction de la Recherche et de l'Innovation, Paris
10301	Friedrich-Baur-Institut	Klopstock, Thomas	Ethik-Kommission der Medizinischen Fakultaet der LMU München
10302	Justus-Liebig-Universitaet Giessen	Lorenz, Birgit	Ethik-Kommission des FB Medizin der Universität Giessen
10303	Universitaetsklinikum Heidelberg	Rohrschneider, Klaus	Ethikkommission der Medizinischen Fakultät Heidelberg
10401	Fondazione IRCCS Istituto Neurologico Carlo Besta Milano	Lamperti, Costanza	Comitato Etico della Fondazione IRCCS Istituto Neurologico "Carlo Besta"
10402	Università di Pisa	Siciliano, Gabriele	Comitato Etico per la Sperimentazione Clinica Area Vasta Nord-Ovest Azienda Ospedaliero-Universitaria Pisana
10403	Azienda Ospedaliera Universitaria Policlinico G. Martino	Toscano, Antonio	Comitato Etico Interaziendale della Provincia di Messina Azienda Ospedaliera Universitaria Policlinico "Gaetano Martino"
10404	G. B. Bietti Fondazione – IRCCS	Parisi, Vincenzo	Comitato Etico Centrale IRCCS - IFO – Fondazione Bietti
10501	Maastricht University Medical Center	Bauer, Noël	METC azM/UM P.
10502	Oogziekenhuis Rotterdam	van Everdingen, Judith	N/A
10702	University Hospital of Wales	Votruba, Marcela	East Midlands - Derby Research Ethics Committee
10703	Moorfields Eye Hospital, London	Yu-Wai-Man, Patrick	North East - Newcastle & North Tyneside 1 Research Ethics Committee
10801	Poznan University of Medical Sciences	Krawczynski, Maciej	Bioethics Committee Poznan University of Medical Sciences

IEC: Independent Ethics Committee; PI: Principal Investigator.

Table S2 Demographics and baseline characteristics of eyes with an available 12- or 24-month assessment and overall eyes.

	12-month analysis			24-month analysis			Overall		
**Disease phase at baseline**	**Subacute/ dynamic**	**Chronic**	**All eyes**	**Subacute/ dynamic**	**Chronic**	**All eyes**	**Subacute/ dynamic**	**Chronic**	**All eyes**
**Characteristic**	n=96	n=11	n=107	n=44	n=2	n=46	n=383	n=19	n=402
Sex, n (%)									
Female	15 (15.6)	3 (27.3)	18 (16.8)	8 (18.2)	2 (100.0)	10 (21.7)	79 (20.6)	3 (15.8)	82 (20.4)
Male	81 (84.4)	8 (72.7)	89 (83.2)	36 (81.8)	0 (0.0)	36 (78.3)	304 (79.4)	16 (84.2)	320 (79.6)
Age at baseline, years									
Mean (± SD)	30.8 (17.4)	30.1 (11.1)	30.7 (16.8)	29.4 (19.6)	20.0 (0.0)	29.0 (19.3)	30.0 (15.2)	28.6 (9.4)	30.0 (15.0)
Median (Q1–Q3)	29.0 (16.0–45.0)	28.0 (20.0–40.0)	29.0 (17.0–43.0)	25.5 (10.0–45.0)	20.0 (20.0–20.0)	24.0 (10.0–45.0)	26.0 (18.0–41.0)	28.0 (20.0–35.0)	26.0 (18.0–41.0)
Min–Max	6.0–65.0	17.0–51.0	6.0–65.0	6.0–64.0	20.0–20.0	6.0–64.0	6.0–68.0	17.0–51.0	6.0–68.0
Variant, n (%)									
m.11778G>A	68 (70.8)	9 (81.8)	77 (72.0)	27 (61.4)	2 (100)	29 (63.0)	273 (71.3)	15 (78.9)	288 (71.6)
m.3460G>A	16 (16.7)	2 (18.2)	18 (16.8)	14 (31.8)	0 (0.0)	14 (30.4)	55 (14.4)	2 (10.5)	57 (14.2)
m.14484T>C	12 (12.5)	0 (0.0)	12 (11.2)	3 (6.8)	0 (0.0)	3 (6.5)	55 (14.4)	2 (10.5)	57 (14.2)
Age at first symptom onset, n (%)									
<15 years	21 (21.9)	0 (0.0)	21 (19.6)	16 (36.4)	0 (0.0)	16 (34.8)	51 (13.3)	0 (0.0)	51 (12.7)
15–35 years	34 (35.4)	8 (72.7)	42 (39.3)	10 (22.7)	2 (100)	12 (26.1)	197 (51.4)	16 (84.2)	213 (53.0)
>35 years[a]	41 (42.7)	3 (27.3)	44 (41.1)	18 (40.9)	0 (0.0)	18 (39.1)	135 (35.2)	3 (15.8)	138 (34.3)
>50 years[a]	16 (16.7)	0 (0.0)	16 (15.0)	9 (20.5)	0 (0.0)	9 (19.6)	47 (12.3)	0 (0.0)	47 (11.7)
Time since first symptom onset at baseline, months									
Mean (± SD)	2.4 (2.7)	25.5 (13.1)	4.8 (8.5)	2.1 (2.4)	19.7 (0.7)	2.9 (4.3)	2.2 (2.3)	22.8 (11.1)	3.1 (5.4)
Median (Q1–Q3)	1.2 (0.4–4.1)	19.3 (16.7–40.0)	1.6 (0.6–5.3)	1.0 (0.4–3.3)	19.7 (19.3–20.2)	1.3 (0.6–3.5)	1.3 (0.6–3.3)	19.3 (15.7–25.5)	1.4 (0.6–3.6)
Min–Max	0.0–10.8	15.7–48.1	0.0–48.1	0.0–10.8	19.3–20.2	0.0–20.2	0.0–10.8	12.8–48.1	0.0–48.1

[a]Some eyes overlap between age groups. Q1–Q3: interquartile range; SD: standard deviation.

Table S3 Demographics and baseline characteristics of eyes subacute/dynamic at baseline (n=383), by sex.

Characteristic	Male<br>n=304	Female<br>n=79
Variant, n (%)		
m.11778G>A	222 (73.0)	51 (64.6)
m.3460G>A	44 (14.5)	11 (13.9)
m.14484T>C	38 (12.5)	17 (21.5)
Age at first symptom onset, years		
Mean (± SD)	27.5 (14.3)	38.9 (15.5)
Median (Q1–Q3)	23.0 (17.0–37.0)	40.0 (28.0–50.0)
Min–Max	6.0–68.0	7.0–65.0
Age at baseline, years		
Mean (± SD)	27.7 (14.3)	39.1 (15.4)
Median (Q1–Q3)	23.0 (17.0–37.5)	40.0 (28.0–50.0)
Min–Max	6.0–68.0	8.0–65.0
Time since first symptom onset at baseline, months		
Mean (± SD)	2.2 (2.2)	2.2 (2.5)
Median (Q1–Q3)	1.3 (0.6–3.3)	1.2 (0.6–2.5)
Min–Max	0.0–10.8	0.0–10.8
Time in observation, months		
Mean (± SD)	32.9 (48.1)	18.7 (33.3)
Median (Q1–Q3)	7.9 (2.0–43.7)	5.7 (2.3–11.5)
Min–Max	0.1–196.4	0.5–135.6

Q1–Q3: interquartile range; SD: standard deviation.

Table S4 VA of eyes subacute/dynamic at baseline in the 12- and 24-month analyses, by sex and age at first symptom onset.

	Male					Female				
	**Overall**	**<15 years**	**15–35 years**	**>35 years**	**>50 years**	**Overall**	**<15 years**	**15–35 years**	**>35 years**	**>50 years**
**12-month analysis**										
**n**	n=81	n=21	n=30	n=30	n=10	n=15	n=0	n=4	n=11	n=6
Baseline VA, logMAR										
Mean (± SD)	1.00 (0.58)	0.76 (0.50)	1.15 (0.64)	1.01 (0.55)	0.92 (0.51)	0.79 (0.56)	-	0.88 (0.75)	0.76 (0.51)	0.64 (0.62)
Median (Q1–Q3)	1.00 (0.52–1.40)	0.70 (0.40–1.00)	1.30 (0.70–1.80)	1.00 (0.60–1.40)	1.00 (0.30–1.30)	0.70 (0.30–1.00)	-	0.85 (0.35–1.40)	0.70 (0.30–1.00)	0.51 (0.30–0.70)
Min–Max	0.00–1.80	0.00–1.80	0.00–1.80	0.20–1.80	0.22–1.80	0.00–1.80	-	0.00–1.80	0.00–1.80	0.00–1.80
12-month VA, logMAR										
Mean (± SD)	1.36 (0.54)	0.94 (0.51)	1.64 (0.39)	1.37 (0.53)	1.18 (0.71)	1.31 (0.64)	-	1.10 (0.54)	1.39 (0.68)	1.80 (0.00)
Median (Q1–Q3)	1.60 (1.00–1.80)	1.00 (0.52–1.10)	1.80 (1.68–1.80)	1.55 (1.00–1.80)	1.55 (0.52–1.80)	1.80 (1.00–1.80)	-	1.05 (0.75–1.45)	1.80 (1.10–1.80)	1.80 (1.80–1.80)
Min–Max	0.00–1.80	0.00–1.80	0.15–1.80	0.15–1.80	0.15–1.80	0.10–1.80	-	0.50–1.80	0.10–1.80	1.80–1.80
Magnitude of VA change from baseline, logMAR										
Mean (± SD)	0.36 (0.60)	0.18 (0.51)	0.49 (0.64)	0.36 (0.60)	0.26 (0.71)	0.52 (0.98)	-	0.23 (1.05)	0.63 (0.98)	1.16 (0.62)
Median (Q1–Q3)	0.30 (0.00–0.70)	0.00 (-0.10–0.49)	0.45 (0.00–0.82)	0.21 (0.00–0.70)	0.25 (-0.15–0.80)	0.80 (0.00–1.40)	-	0.60 (-0.45–0.90)	1.10 (0.00–1.50)	1.29 (1.10–1.50)
Min–Max	-0.78–1.80	-0.78–1.12	-0.54–1.80	-0.78–1.60	-0.78–1.50	-1.30–1.80	-	-1.30–1.00	-0.90–1.80	0.00–1.80
**24-month analysis**										
**n**	n=36	n=14	n=10	n=12	n=5	n=8	n=2	n=0	n=6	n=4
Baseline VA, logMAR										
Mean (± SD)	0.94 (0.56)	0.63 (0.34)	1.29 (0.49)	1.00 (0.65)	0.76 (0.52)	0.99 (0.66)	1.65 (0.21)	-	0.77 (0.61)	0.78 (0.69)
Median (Q1–Q3)	0.89 (0.50–1.30)	0.60 (0.40–1.00)	1.30 (0.70–1.80)	1.04 (0.30–1.64)	0.70 (0.30–1.30)	0.92 (0.35–1.65)	1.65 (1.50–1.80)	-	0.51 (0.30–1.22)	0.51 (0.35–1.21)
Min–Max	0.00–1.80	0.00–1.00	0.70–1.80	0.20–1.80	0.22–1.30	0.30–1.80	1.50–1.80	-	0.30–1.80	0.30–1.80
24-month VA, logMAR										
Mean (± SD)	1.00 (0.66)	0.73 (0.42)	1.39 (0.59)	1.00 (0.80)	0.31 (0.67)	1.57 (0.37)	1.00 (0.14)	-	1.75 (0.11)	1.80 (0.00)
Median (Q1–Q3)	1.00 (0.46–1.66)	0.70 (0.40–1.00)	1.66 (1.00–1.80)	1.30 (0.02–1.80)	0.00 (0.00–0.05)	1.80 (1.31–1.80)	1.00 (0.90–1.10)	-	1.80 (1.80–1.80)	1.80 (1.80–1.80)
Min–Max	0.00–1.80	0.00–1.52	0.00–1.80	0.00–1.80	0.00–1.52	0.90–1.80	0.90–1.10	-	1.52–1.80	1.80–1.80
Magnitude of VA change from baseline, logMAR										
Mean (± SD)	0.06 (0.67)	0.09 (0.46)	0.10 (0.65)	-0.00 (0.92)	-0.45 (0.88)	0.57 (0.94)	-0.65 (0.07)	-	0.98 (0.66)	1.02 (0.69)
Median (Q1–Q3)	0.00 (-0.26–0.29)	0.00 (-0.1–0.18)	0.11 (0.00–0.30)	-0.18 (-0.40–0.41)	-0.30 (-1.26– -0.22)	0.74 (-0.30–1.45)	-0.65 (-0.70– -0.60)	-	1.29 (0.30–1.50)	1.29 (0.59–1.45)
Min–Max	-1.30–1.60	-0.48–1.12	-1.30–1.10	-1.30–1.60	-1.30–0.82	-0.70–1.50	-0.70– -0.60	-	0.00–1.50	0.00–1.50

logMAR: logarithm of the minimum angle of resolution; Q1–Q3: interquartile range; SD: standard deviation; VA: visual acuity.

Table S5 R^2 and type 3 tests on linear regressions of patient and disease variables for change in VA in the 12- and 24-month analyses of eyes subacute/dynamic at baseline.

12-month analysis				24-month analysis			
	Model	Type 3 test			Model	Type 3 test	
R^2	Variable	F-statistic	p-value	R^2	Variable	F-statistic	p-value
0.47	Baseline VA	65.4	<0.0001	0.51	Baseline VA	12.4	0.0011
	Variant	8.7	0.0004		Variant	9.1	0.0005
0.46	Baseline VA	74.7	<0.0001	0.38	Baseline VA	22.4	<0.0001
	Age group at first symptom onset	7.8	0.0008		Age group at first symptom onset	3.1	0.0566
0.37	Baseline VA	54.1	<0.0001	0.37	Baseline VA	19.6	0.0001
	Sex	0	0.9079		Sex	5.4	0.0251

VA: visual acuity.

Fig. S1 Proportion of patients (N=219) with either one eye (sequential onset) or both eyes (simultaneous onset) affected within 1 month of first symptom onset, and time between symptom onset in sequential eyes by (a) causative mtDNA variant, (b) age at first symptom onset, and (c) sex. *Out of 119 patients who had one eye affected at the onset of symptoms, 8 patients did not develop symptoms in their second eye during the observation period (median [Q1–Q3]; 14.3 [1.9–61.9] months) and were therefore excluded from the Δ symptom onset calculation. mtDNA: mitochondrial DNA; Q1–Q3: interquartile range; SD: standard deviation.

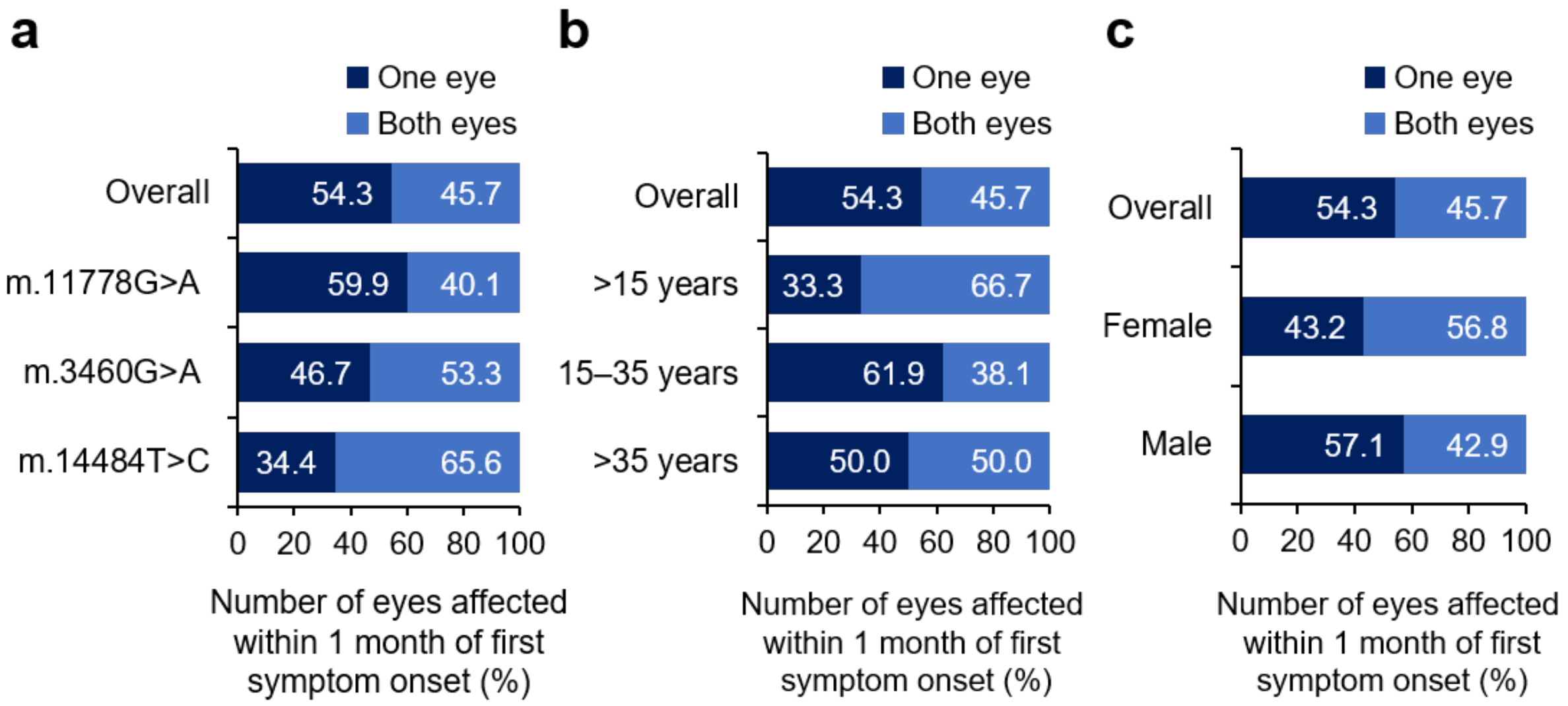

Δ symptom onset between eyes (months)

	N*	Mean (SD)	Median (Q1–Q3)
Overall	111	2.9 (2.4)	2.0 (1.0–4.0)
m.11778G>A	87	2.9 (2.4)	2.0 (1.0–4.0)
m.3460G>A	14	2.6 (1.5)	2.6 (1.0–3.9)
m.14484T>C	10	3.0 (3.4)	1.5 (1.0–4.0)

	N*	Mean (SD)	Median (Q1–Q3)
Overall	111	2.9 (2.4)	2.0 (1.0–4.0)
<15 years	7	4.7 (4.3)	5.8 (0.5–7.0)
15–35 years	70	2.7 (2.5)	2.0 (1.0–4.0)
>35 years	34	2.7 (1.3)	2.9 (2.0–3.6)

	N*	Mean (SD)	Median (Q1–Q3)
Overall	111	2.9 (2.4)	2.0 (1.0–4.0)
Male	94	2.8 (2.4)	2.0 (1.0–3.9)
Female	17	3.0 (2.2)	2.8 (1.0–4.0)

Fig. S2 Clinical outcome rates by sex in eyes subacute/dynamic at baseline in the (**a**) 12- and (**b**) 24-month analyses. *Only those eyes eligible for these definitions at baseline were used to calculate the proportion. sCRB: spontaneous clinically relevant benefit; sCRR: spontaneous clinically relevant recovery; sCRS: spontaneous clinically relevant stabilization; sCRW: spontaneous clinically relevant worsening.

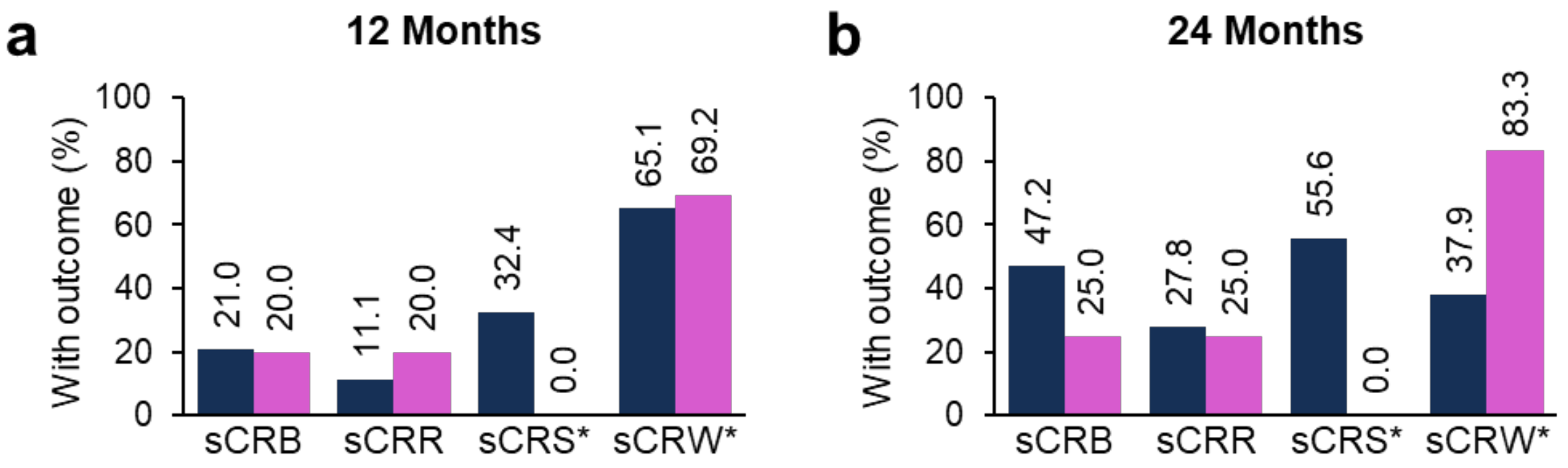

With outcome / eligible eyes (n/N)				
Male	17/81	9/81	11/34	41/63
Female	3/15	3/15	0/8	9/13

With outcome / eligible eyes (n/N)				
Male	17/36	10/36	10/18	11/29
Female	2/8	2/8	0/4	5/6

Fig. S3 Cumulative proportion curves of eyes subacute/dynamic at baseline that showed (**a**) sCRR (n=67) or (**b**) sCRW (n=188) at their last assessment, by sex. Curves are shown over a period of 24 months. *Only those eyes eligible for sCRW (i.e., those eyes on-chart at baseline) are shown in the denominator. Q1–Q3: interquartile range; sCRR: spontaneous clinically relevant recovery; sCRW: spontaneous clinically relevant worsening.

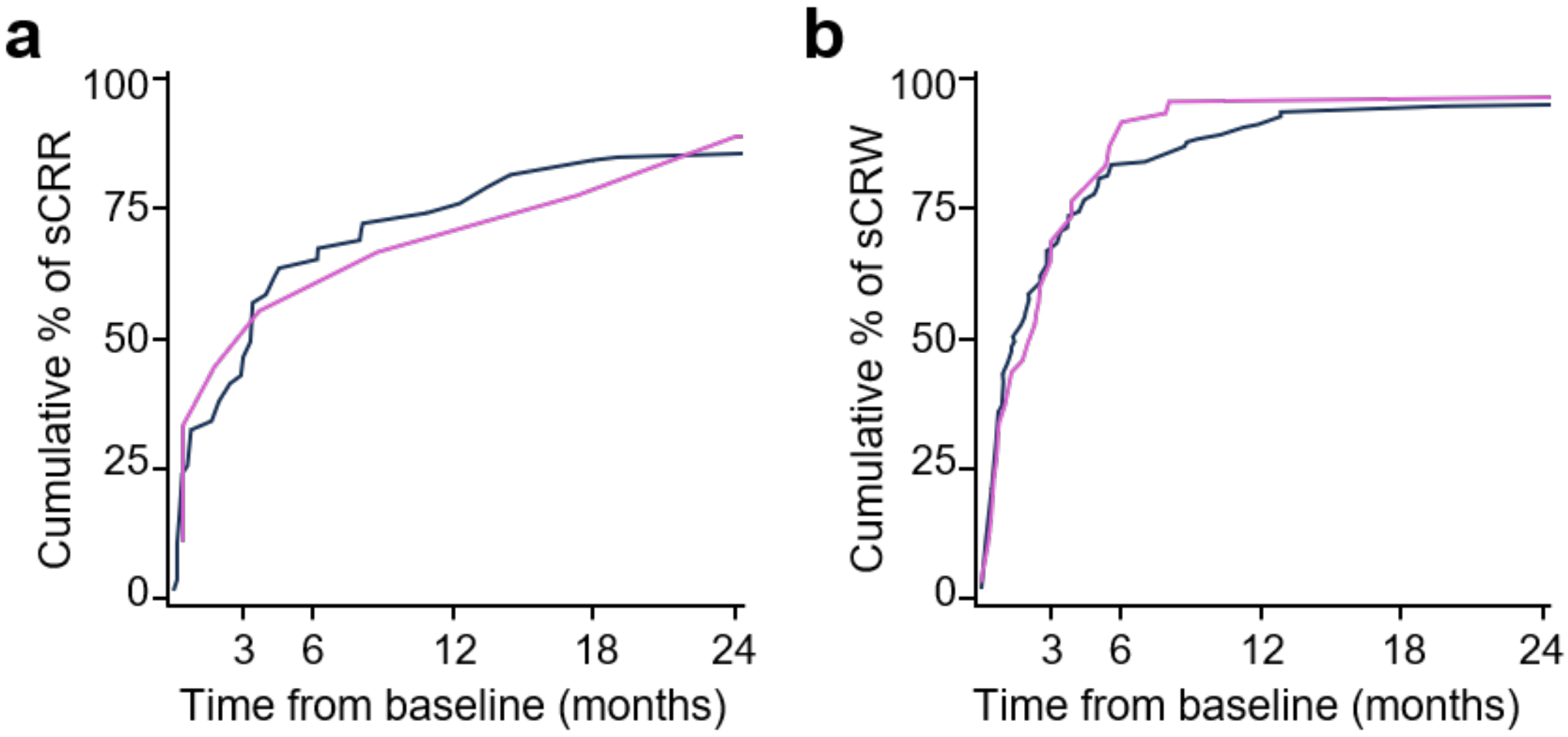

Time to sCRR (months)		
	n/N	Median (Q1–Q3)
Male	58/304	3.4 (0.6–12.2)
Female	9/79	3.7 (0.5–17.2)

Time to sCRW (months)		
	n/N*	Median (Q1–Q3)
Male	140/224	1.4 (0.5–4.3)
Female	48/69	2.2 (0.7–3.9)

Fig. S4 Clinical outcome rates of eyes subacute/dynamic at baseline for all eyes with an available VA assessment, by causative mtDNA variant, using outcome thresholds of ±0.2 logMAR and ±0.3 logMAR in the (**a**) 12-month and (**b**) 24-month analyses. *Only those eyes eligible for sCRW at baseline were used to calculate the sCRW proportions. mtDNA: mitochondrial DNA; sCRR: spontaneous clinically relevant recovery; sCRW: spontaneous clinically relevant worsening; VA: visual acuity.

a

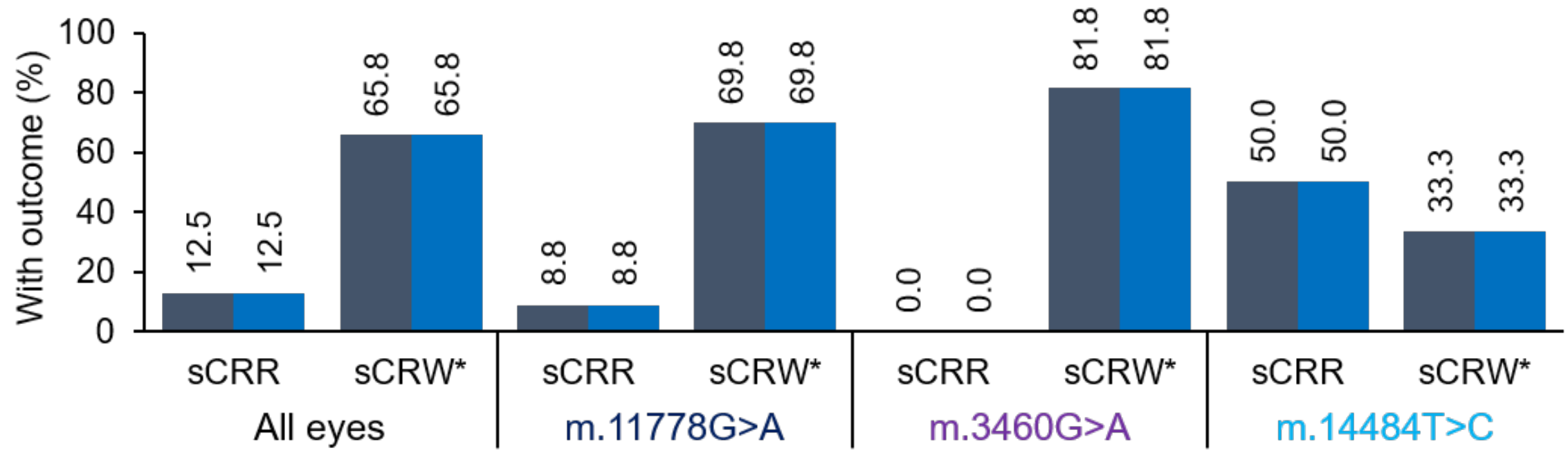

With outcome / eligible eyes (n/N)								
0.2 logMAR	12/96	50/76	6/68	37/53	0/16	9/11	6/12	4/12
0.3 logMAR	12/96	50/76	6/68	37/53	0/16	9/11	6/12	4/12

b

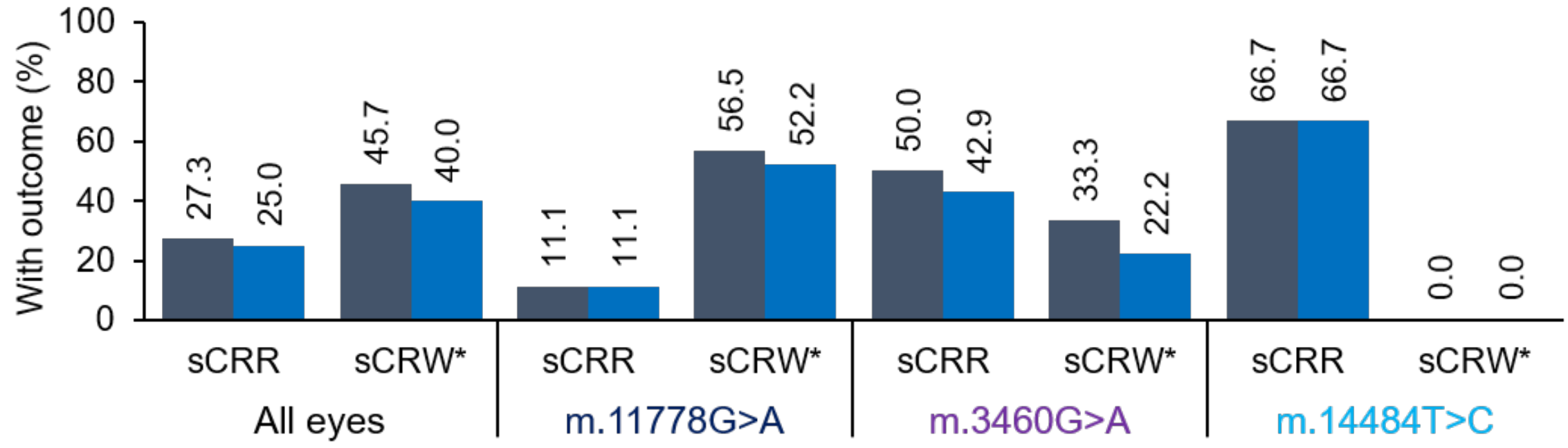

With outcome / eligible eyes (n/N)								
0.2 logMAR	12/44	16/35	3/27	13/23	7/14	3/9	2/3	0/3
0.3 logMAR	11/44	14/35	3/27	12/23	6/14	2/9	2/3	0/3
